# Phage Immunoprecipitation and Sequencing—a Versatile Technique for Mapping the Antibody Reactome

**DOI:** 10.1016/j.mcpro.2024.100831

**Published:** 2024-08-19

**Authors:** Gustav N. Sundell, Sheng-Ce Tao

**Affiliations:** Shanghai Center for Systems Biomedicine, Key Laboratory of Systems Biomedicine (Ministry of Education), Shanghai Jiao Tong University, Shanghai, China

**Keywords:** phage display, PhIP-seq, antigen discovery, biomarker, autoimmunity

## Abstract

Characterizing the antibody reactome for circulating antibodies provide insight into pathogen exposure, allergies, and autoimmune diseases. This is important for biomarker discovery, clinical diagnosis, and prognosis of disease progression, as well as population-level insights into the immune system. The emerging technology phage display immunoprecipitation and sequencing (PhIP-seq) is a high-throughput method for identifying antigens/epitopes of the antibody reactome. In PhIP-seq, libraries with sequences of defined lengths and overlapping segments are bioinformatically designed using naturally occurring proteins and cloned into phage genomes to be displayed on the surface. These libraries are used in immunoprecipitation experiments of circulating antibodies. This can be done with parallel samples from multiple sources, and the DNA inserts from the bound phages are barcoded and subjected to next-generation sequencing for hit determination. PhIP-seq is a powerful technique for characterizing the antibody reactome that has undergone rapid advances in recent years. In this review, we comprehensively describe the history of PhIP-seq and discuss recent advances in library design and applications.

## Highlights

• Comprehensive overview of PhIP-seq library creation and published PhIP-seq libraries.
• Overview of statistical strategies of PhIP-seq data analysis.
• Applications of PhIP-seq for analyzing the antibody reactome in health and disease.
• Drawbacks of PhIP-seq and future perspectives.

## Graphical Abstract

**Figure:**
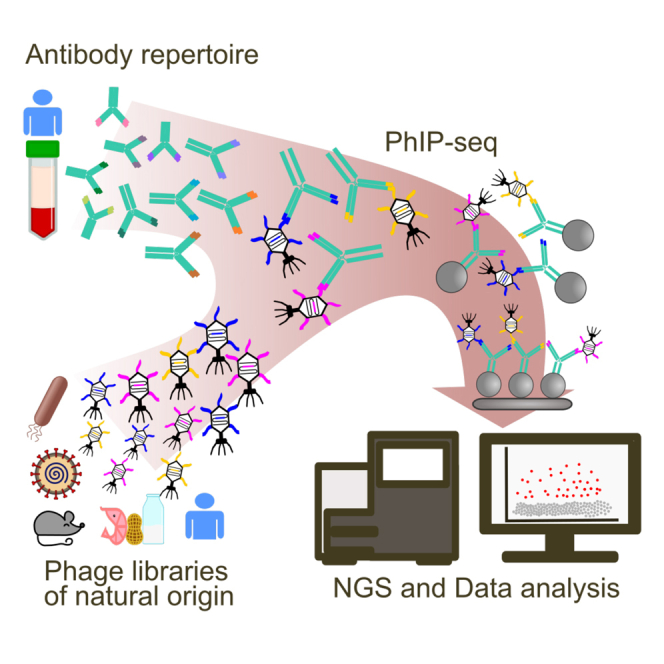

## In Brief

Phage immunoprecipitation and sequencing (PhIP-seq) investigates the antibody reactome by using phage-displayed peptide libraries of proteomic origin to map antibody reactivity and aims to answer fundamental questions about the immune system as well as providing tools for biomarker discovery and investigating pathology for infectious diseases, autoimmune diseases, and allergy. This review highlights library design and production and investigative uses of this technology.

Determining the antibody reactome, that is, what antigens an antibody repertoire are binding to can reveal current or prior infection (1, 2), what a person is allergic to (3), and antibodies reacting to proteins from one’s own body that can cause or contribute to autoimmune diseases (4, 5). This can be used for understanding the disease, which can lead to better treatment (6), determining how the immune system responds to prior infection (7), determining candidates for rational vaccine design (8), finding biomarkers for certain diseases (9), including biomarkers that distinguish disease severity or progression (10), and ultimately adding to greater understanding of the immune system (11). Every individual has a personal antibody reactome consisting of antibodies that recognize epitopes that are more unique to that person, these are called private epitopes, and epitopes that are shared between individuals (public epitopes) (12). To determine this information, it is necessary to test a subject’s repertoire of antibodies to a set of peptides or proteins to determine what the antibodies bind to. Historically, this has been done through peptide/protein array and serological proteome analysis (SERPA). SERPA is a method in which a set of different yet unknown proteins from cell lysates are separated by 2D-PAGE. This is then transferred to a Western blot membrane and incubated with serum to allow for antibody binding to the antigens. Bound antibodies are then detected by standard Western blot techniques. After that, positive spots can be excised from a separate protein gel and subjected to mass spectrometry to determine what protein(s) were present in the spot (13). The advantage of this method is that it uses proteins that carry normal posttranslational modifications. In native gels, proteins maintain their structure, so antibody–antigen interactions based on structural and linear epitopes can be identified; this method is easily accessible for most laboratories and has a relatively low cost. Moreover, because these methods are based on lysates from growing cells, tissues or bacteria, the relative abundances of individual proteins are different, which means that highly abundant but less reactive proteins can be identified over less abundant but more reactive proteins. There is a limited supply of lysates, which makes it difficult to analyze more than a few serum samples based on the same lysate, and antibody–antigen interactions are not resolved at the epitope level (14).

Another more classical technique is peptide and protein arrays, where peptides are synthesized either in an array format on an array or off array and then immobilized on an array, or proteins are produced and attached to arrays, which leads to spots on the arrays with defined proteins or peptides. These arrays are then incubated with serum and subsequent fluorescent secondary antibodies and analyzed. This provides an instant readout of which peptides or proteins are immune reactive. Protein microarrays can detect interactions based on structural and linear epitopes (15, 16), while peptide microarrays can detect antibody–antigen interactions at the linear epitope level (17). Microarrays of proteomes of various infectious diseases have been used for antigen identification (15, 16)^,^ and human protein microarrays have been used to identify biomarkers for cancer (18) and autoimmune diseases (19, 20). The main disadvantages of protein and peptide arrays are cost and size. To construct protein arrays, many proteins of interest need to be expressed and purified. Producing vectors containing the ORFs of interest for recombinant expression in *Escherichia coli*, yeast or insect cells, subsequent affinity tag purification, and spotting on the arrays can take a long time to optimize and be a costly process, and some proteins might still not be able to be expressed; this includes some membrane proteins that are usual targets for antibodies. It is also difficult to determine the proper folding of proteins on an array. The most extensive commercial protein microarray can display between 20,000 and 30,000 proteins (20), and peptide microarrays can display over two million peptides on a single array (21). Peptide arrays usually have significant overlap of the tiling between peptides to avoid breaking up epitopes and false positives (22). Each array can only be used for one sample, scaling the cost for multiple samples quickly.

Phage-based techniques have emerged as alternatives to SERPA and protein/peptide microarrays. The phage species used in phage displays are viruses with protein envelopes derived from the ORFs of the phage. By modifying the genome of the phage to display a protein or peptide as a fusion protein, one of the phage coat proteins on the surface can be achieved. This genotype-to-phenotype link makes it possible to use DNA sequencing to determine what protein or peptide is displayed. Phage display was first described by GP Smith in 1985 (23), where he inserted a 171 bp long DNA fragment coding for parts of the EcoRI endonuclease gene into the pIII gene of the filamentous phage f1 and managed to amplify it compared to the background M13 phage by binding it with an antibody, and he postulated that using a library of random inserts could be used in selection (23). Phage display has the advantage that after library production, the same library can be used in thousands of selections, decreasing the cost per serum sample tested. In the mid-2000s, phage display began to be used for antigen discovery, where complementary DNA (cDNA) from patients was cloned and inserted into vectors accounting for the three different translation frames to create libraries of 10^6^ theoretical library sizes and used in standard phage display experiments with serum from patients immobilized on the surface as bait, with serum from healthy people used as preselection. Through several rounds of selection with amplification steps in bacteria (24), individual clones from the selection were picked through enzyme-linked immunosorbent assay (24) or through incorporation into a protein microarray (25, 26), and positive clones were sequenced through Sanger sequencing. This approach has been used to find antigens for autoantibodies and disease-specific antibodies from serum (24, 25, 26). It can also detect antigen–antibody interactions based on both structural and linear epitopes, although information about the specific epitope is lacking. One of the disadvantages of cDNA display is that a large portion of the library contains out-of-frame proteins, in some cases as much as 94% of the library (27). Another disadvantage is that shorter proteins tend to outgrow longer proteins, which can lead to unintended bias in selection, especially after several rounds (27, 28). The lengths of the inserts make them incompatible with next-generation sequencing. In the 2010s, a novel phage display method for immunoproteomics called phage immunoprecipitation and sequencing (PhIP-seq) was developed to address the problems associated with cDNA display (4).

## Phage Immunoprecipitation and Sequencing

**Fig. 1:**
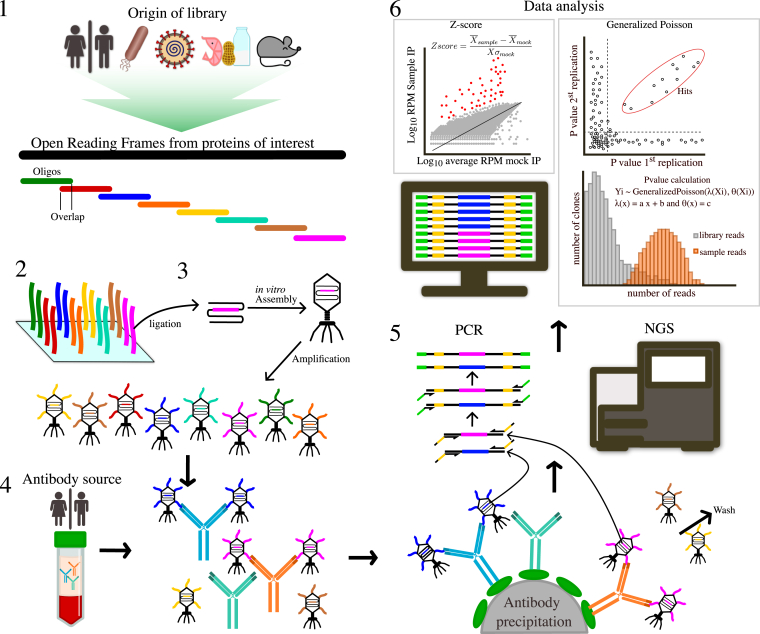
**Schematic representation of PhIP-seq.** Library design and synthesis, PhIP-seq selection, next-generation sequencing, and statistical analysis. *1*, the ORFs of the proteins of interest were bioinformatically extracted and segmented into peptides of equal length with an overlap to avoid splitting epitopes. *2*, the peptide sequences were reverse translated into DNA to produce an oligonucleotide pool. *3*, incorporate the DNA *via* ligation, packing, and amplification to pair particles expressing the peptides on the surface. *4*, preform selection by mixing phage and serum/plasma/CSF and pull down with magnetic beads binding of the antibodies and washing away unbound phages. *5*, PCR products were used to amplify and barcode the sequences, which were subsequently subjected to next-generation sequencing. Specific barcodes for demultiplexing the sequences to the right selection. *6*, statistical analysis for hit identification and subsequent data analysis. CSF, cerebrospinal fluid; PhIP-Seq, phage display immunoprecipitation and sequencing.

PhIP-seq is a novel powerful technique that uses well-defined proteomic libraries of equal length to probe circulating antibodies and pull-down interacting partners by immunoprecipitation (IP). After precipitation, the phages are subjected to next-generation sequencing. In this section, we describe the library design of published PhIP-seq libraries, the creation of T7 phage libraries, PhIP-seq selection, and hit determination (Fig. 1). The rapid advances in PhIP-seq technology in the past few years warrant comprehensive review. Though there have been previous reviews has had its focus on specific libraries and applications like viral infectious diseases (29) or food antigens (30) together with a general overview of library design and statistical analysis. To our knowledge, this review describes all the PhIP-seq libraries published to date, including library design, production, selection, and hit determination. We address many aspects of how different studies have utilized the PhIP-seq method, especially how the technique has been used together with machine learning, and discuss current limitations and future perspectives of PhIP-seq technology.

### PhIP-Seq Libraries and Their Design

The first step of a PhIP-seq experiment is based on a scientific question and the selection of a library suitable for answering the question. In classical phage display, randomized libraries are typically constructed by saturation mutagenesis PCR using degenerate NNK or NNS codons (where N = A/C/G/T, K = G/T and S = C/G) (31, 32). Around 2010, large-scale DNA microarrays have become precise enough and inexpensive enough for the production of large oligonucleotide libraries by rational design. This enabled Larman *et al.* (4) to produce a library representing the entire human proteome in 2011 and use it to perform IP experiments. In this section we will go through all different types of PhIP-seq libraries that have been published based on origins of the library. To represent all the possible shorter epitopes for antibody binding, it is not enough to partition the proteins because this runs the risk of splitting an epitope between two peptides or placing the epitope so close to the end of the peptide that it is functionally inaccessible for the antibody to bind. Therefore, tiling the proteome with substantial overlap between the peptides is desirable to avoid this issue.

### Human Peptidomic Libraries

The library produced by Larman *et al*. was constructed from the 24,239 unique ORFs of the 35.1 built-up human genome, the authors divided the proteins into 36 aa residue-long peptides (36-mer) with an overlap of seven residues, which resulted in a theoretical library of 413,611 peptides from the human proteome (Table 1). Sequencing of the library had a coverage of peptides of 91.8%, and the library was fairly uniform, with 78% of the library members within an order of magnitude from the mean peptide (4). This research group also constructed a 90-mer and 45-residue overlapping human peptidome library consisting of 274,207 peptides first used in Xu *et al*. 2016 (33), but further details regarding library composition, coverage, and quality have never been described for this library. In 2020, the research group of DeRisi published their own version of a human peptidome library in O’Donovan *et al.* (34). This library consists of all human sequences, including splicing isoforms of proteins and predicted sequences, in the NCBI protein database as of November 2015, which means that it includes proteins and splice variants not present in the Larman *et al*. library, but potentially peptides from putative proteins that never gets translated. This library is divided into 49-mer peptides with 24 overlapping residues; this resulted in a library of 731,724 unique peptides (Table 1).

**Table 1:** Design and content of published PhIP-seq libraries and where they were used

Lead author	Origin of library	Peptide length and overlap	Number of peptides	Also used
Larman et al. 2011 (4) and Xu et al. 2016 (33)	24,239 unique ORFs from build 35.1 of the human genome	36-mer peptides, 7-aa overlap and 90-mer peptides 45-aa overlap	413,611 peptides and 274,207 peptides	(5, 50, 60, 81, 105, 106, 110, 111, 129, 130)
Xu et al. 2015 (2)	All viruses with human tropism in the UniProt database. Includes 206 viruses and over 1000 different strains	56-mer peptides, 28-aa overlap	93,904 peptides	(11, 40, 49, 50, 55, 59, 61, 68, 70, 74, 76, 90, 91, 92, 93, 96, 97, 99, 107, 113, 131, 132)
Mina et al. 2019 (66)	Updated VirScan from Xu et al.18 new viruses plus antigens from the Immune Epitope Database that are not from viruses or malaria (mostly bacterial immune epitopes)	56-mer peptides, 28-aa overlap	115,753 peptides	(63, 80, 89, 94, 95, 108, 114, 133, 134, 135, 136, 137)
Schubert et al. 2019 (35)	Viral genomes infecting vertebrate, mosquito-borne, and tick-borne downloaded from the UniProt/RefSeq in Feb 2017	62-mer peptides, 14-aa overlap	481,966 peptides	(1, 36, 86, 98)
O’Donovan et al. 2020 (34)	All human sequences in the NCBI protein database including with splicing isoforms and predicted coding regions. (Nov 2015)	49-mer peptides, 25-aa overlap	731,724 peptides	(50, 58, 65, 67, 69, 72, 73, 75, 83, 84, 85, 86, 87, 102, 104, 138, 139)
Zamecnik et al. 2020 (36)	Two libraries: 1) SARS-CoV-2–specific and 2) seven known hCoVs, including SARS-CoV-1 and SARS-CoV-2, downloaded from NCBI	38-mer peptides, 19-aa overlap	534 and 3670 peptides, respectively	(100, 101)
Shrock et al. 2020 (37)	Three libraries two for SARS-CoV-2 one covering SARS-CoV-2 variant sequences available early in the pandemic, one triple alanine scan, and one for six hCoVs and three bat coronaviruses	20-mer and 5-aa overlap SARS-CoV-2–specific library, 56-mer 28-aa overlap for CoV species and triple alanine scan libraries	4288 in 20-mer SARS-CoV-2 library, 6932 peptides in 56-mer CoV library and 47,557 peptides in alanine scan library	(61, 79, 132, 140)
Klompus et al. 2021 (39)	Seven hCoVs downloaded from NCBI and variants of the SARS-CoV-2 ORF reported mid-April of 2020 and with 49 animal CoV strains	64-mer peptides with 20-aa overlaps	13,192 peptides	
Stoddard et al. 2021 (38)	17 different CoV strains four common hCoV plus SARS-CoV and MERS-CoV SARS-CoV-2 and bat SL-CoVZC45	39-mer peptides, 19-aa overlap	10,047 peptides	(57)
Monaco et al. 2021 (45)	All protein sequences from UniProt database included in the Allergome	56-mer peptides, 28-aa overlap	19,331 peptides	
Rajan et al. 2021 (41)	All protein sequences from 74 flaviviruses that infects human (NCBI)	62-mer peptides, 54-aa overlap	91,562 peptides	
Vogl et al. 2021 (48)	Potential antigens from the microbiome of 953 stool samples plus pathogenic bacteria, probiotic bacteria, and gut microbiota reported to be coated by antibodies plus sequences from the virulence factor database. Total 28,668 proteins, 27,837 of them from microbiota	64-mer peptides, 20-aa overlap	244,000 peptides	(47, 64)
Venkataraman et al. 2022 (40)	Sequences from internal sequencing and publicly available anellovirus genomes from the NCBI GenBank repository total 834 anellovirus	56-mer peptides, 28-aa overlap	32,96 peptides	
Angkeow et al. 2022 (49)	Nonviral proteins from UniProt with ‘‘toxin’’ or ‘‘virulence factor’’ keyword associations (Nov 2016). The resulting 14,430 protein sequences (436 archaeal, 7797 bacterial, and 5743 eukaryotic)	56-mer peptides, 28-aa overlap	95,601 peptides	
Leviatan et al. 2022 (46) and Bourgonje et al. 2023 (47)	Library includes five allergen databases: Allergome, World Health Organization (WHO)/International Union of Immunological Societies Allergen Nomenclature Database, Structural Database of Allergenic Proteins, AllergenOnline, and the database of allergen families, plus the full list of B-cell antigens deposited in the IEDB. And around 40,000 sequences from phages infecting gut bacteria	54-mer peptides, 20-residue overlap	58,233 peptides or allergenic and IEDB peptides and around 40,000 gut phage peptides leading to a library size of around 100,000 peptides	(47, 64)
Rasquinha et al. 2022 (50)	Mouse proteome (UP000000589) from the UniProt database (accessed on 1 Jan 2018)	56-mer peptides, 28-residue overlap	419,915 peptides	
Raghavan et al. 2023 (44)	Plasmodium falciparum reference proteomes 3D7, IT plus variant antigens total 7577 plasmodium proteins	62-mer peptides, 25-residue overlap	238,068 peptides	
Chen et al. 2021 (3)	12 peanut allergens and saturation mutagenesis	20-mer peptides, 10-residue overlap	397 peptides underwent saturation mutagenesis, total 149,128 peptides	
Rackaityte et al. 2023 (51)	*Mus musculus* reference proteome (GRCm38.p5), including all isoforms (GRCm38.p5) from NCBI	62-mer peptides, 19-aa overlap	482,672 peptides	
Yaffe et al. 2023 (43)	The envelope protein from seven strains of HIV	38-mer peptides 36-aa overlap	1369 peptides	
Liebhoff et al. 2024 (https://zenodo.org/records/10448728)	112 species present in samples from North America in the Gut phage database (GPD) was used to create three different repertoires in the library one “normal” set with long overlapping peptides using pepsyn, two libraries where machine learning algorithm Dolphyn was used to decrease the sequence space to potential antigen epitopes.	Subset 1: 56-mer peptides, 28-residue overlap; subset 2: 15-mer peptides representing the Dolphyn predicted epitopes; subset 3: 56-mer peptides containing three sequential Dolphyn predicted epitopes with a GGGGS linker between	subset 1, 23,745 subset 2, 19,117, subset 3, 5266 total 48,128 peptides	
Woellner-Santos et al. 2024 (8)	1641 proteins from all *Schistosoma mansoni* life-cycle stages	58-mer peptides 7-aa overlap	119,747 peptides	
Morgenlander et al. 2024 (42)	691 arbovirus species’ genomes from 20 different taxonomies of possible Zoonotic viruses from arthropods resulting in 25,138 proteins	1 56-mer peptides, 28-residue overlap	210,976 peptides	
Wu et al. 2019 (9)	Ph.D-12 randomized phage library	12-mer peptides	Theoretically 10^9^ sequences	

IEDB, Immune Epitope Database; MERS-CoV, Middle East respiratory syndrome corona virus; PhIP-Seq, phage display immunoprecipitation and sequencing.

### Pan Viral Libraries

In 2015, Xu *et al*. (2) developed the VirScan library. This library, instead of originating from a single organism proteome, consists of peptides from multiple human viral pathogens. The origin sequences of this library were viruses annotated to have human tropism downloaded from the UniProt database. This library consists of 56-mer peptides and a 28-residue overlap which means both longer peptides and more overlapping residues than the human peptidome library previously published by the same group (4). The library consists of 93,904 peptides from proteins in over 1000 pathogenic strains of 206 viral species (Table 1). In 2019, this group extended the VirScan library with 18 more virus strains (*e.g.*, Zika virus and MERS) and all reported antigens from the Immune Epitope Database (IEDB) that does not have human, viral, or malarial origin, resulting in immune epitopes that are primarily from bacteria. A total of 115,753 peptides were identified in this VirScan 2.0 library, as described in the 2019 paper by Mina *et al.* (66). In 2019, Schubert *et al*. (35) constructed a separate virus library that is significantly larger than that from Mina *et al*. with a total of 481,966 peptides from virus sequences from the UniProt and RefSeq databases downloaded in Feb. 2017, excluding viruses that have algae, archaea, diatom, bacteria, environment, fungi, plants as hosts, and all giant viruses. Clinically relevant viruses from 19 species not included in the databases were added along with vaccine strains from an additional five viruses. The library was divided into 62-mer peptides with a 14-residue overlap.

### Corona Virus Libraries

In 2020 and 2021, a number of papers with describing different coronavirus-specific libraries were reported. Zamecnik *et al.* (36) describes two libraries, one severe acute respiratory syndrome coronavirus 2 (SARS-CoV-2)–specific library and one library including the seven coronaviruses infecting humans, including Middle East respiratory syndrome corona virus, SARS-CoV, and SARS-CoV-2. The libraries included 38-mer peptides with 19 overlapping residues and 534 and 3670 peptides, respectively. Shrock *et al.* (37) made three separate libraries, one for SARS-CoV-2–specific, covering SARS-CoV-2 variant sequences available early in the pandemic, one library with a triple alanine scan of the SARS-CoV-2 sequences meaning three consecutive residues of the peptide substituted with alanine and then repeated throughout the peptide, and finally one for six human coronaviruses and three bat coronaviruses. The SARS-CoV-2–specific library contains 20-mer peptides with five overlapping residues, and the other libraries contain 56-mer peptides with 28 overlapping residues, which resulted in 6932 peptides in the 56-mer CoV library, 47,557 peptides in the triple alanine scan SARS-CoV-2 library, and 4288 in the 20-mer SARS-CoV-2 library. Stoddard *et al*. (38) created a library of several strains of the four common hCoVs plus Middle East respiratory syndrome corona virus SARS-CoV and SARS-CoV-2 plus one bat CoV, the peptides were divided in to 39-mer peptides with 19 aa residue overlap. The total size of the library was 10,047 peptides. Finally, Klompus *et al*. (39) constructed a library including seven human coronaviruses, including SARS-CoV-2, together with 49 animal CoV strains divided into 64-mer peptides with 20-residue overlaps, resulting in a library of 13,192 peptides.

### Libraries from Specific Pathogens

VirScan contains hundreds of viruses which means that not all variants of the viruses can be included instead it is usually restricted to reference genomes. By making dedicated libraries for virus classes makes it easier to include more variants of the viruses, as was done for the coronavirus-specific libraries. This strategy has been done for other pathogens as well. Anelloscan published by Venkataraman *et al*. (40) In 2020, anelloviruses, which are a class of viruses that are commensal to the human body, were studied, which means that it establishes itself as a chronic infection in the body. Anelloviruses are present in 90% of the human population. These viruses have a high sequence diversity, and all humans have their own specific history of infection, leading to a unique personal anellome (40). The PhIP-seq library consists of ORFs from 400 alpha beta and gamma anelloviruses split into 56-mer peptides, with 28 residues overlapping for a total of 32,960 peptides. Rajan *et al*. 2021 produced a flavivirus-specific library consisting of 74 flavivirus species known to infect humans downloaded from NCBI in November of 2017. The proteins were tiled into 62-mer peptides with a substantial 54 aa residue overlap, resulting in a library of 91,562 peptides. They used this library to identify antibody reactivity after dengue virus infection in 20 nonhuman primates at different timepoints after infection (41). Recently Morgenlander *et al*. presented a library of arboviruses, this is not a specific class of viruses instead it is a group of viruses that comes from an arthropod vector like ticks or mosquitoes. This includes viruses like Dengue virus which was the focus of Rajan *et al*. (41) but also viruses like Zika virus. In total, the library consists of 691 arbovirus species’ genomes curated by the authors from different sources and the sequences were downloaded from GenBank in 2017. The final library includes 210,976 peptides of length 56 aa with 28 aa overlap (42). Yaffe *et al*. (43) made a library from the human immunodeficiency virus (HIV) envelope protein from seven strains derived from participants in the study. The library was 1369 peptides large and consisted of 38-mer long peptides with 36 aa overlaps. Raghavan *et al.* (44) constructed a library of *Plasmodium falciparum*, the parasite causing malaria. The library consists of 3D7 and IT reference proteomes plus variant antigens, 7577 plasmodium proteins, and some proteins from Anopheles salivary proteins (the mosquito vector) split into 62-mer peptides with 25 residues overlapping, resulting in 238,068 peptides from 8980 proteins. Woellner-Santos *et al*. (8) presented a library of the parasite *schistosomiasis mansoni*, where they tiled V5.2 of the *Schistosoma mansoni* transcriptome in 56-mer long peptides with seven residue overlaps, resulting in an 119,747-sequence large library. This library differs from other PhIP-seq libraries by using M13 phage and not T7 to construct the library. One of the arguments in Larman’s original PhIP-seq paper for using T7 is that the physical phage library should yield less sequence bias and be more uniform due to differences in virus packing (4); however, in this paper, they show that, through the total of next generation sequencing (NGS) performed that the coverage is good at 99.6%, even though they do not answer the question of how uniform the input library is (8).

### Allergen Libraries

Monaco *et al.* (45) used the curated Allergome database downloaded from UniProt (August 2017) and split 1847 allergenic proteins into 56-mer peptide tiles with 28 residue overlaps, resulting in a library of 19,331 peptides. Chen *et al*. (3) made a peanut-specific allergen library using 12 peanut allergens and saturation mutagenesis (substitution of a single amino acid to every other amino acid) divided into 20-mer peptides with ten overlapping residues. The 397 initial peptides were subjected to saturation mutagenesis, generating 149,128 total peptides. Leviatan *et al*. (46) constructed a library of food and environmental allergens using five allergen databases, the Allergen Nomenclature Database, Allergome, AllergenOnline, the AllFam database and the structural database of allergenic proteins. They took the full-length proteins and split them in to peptides of 54-mer length with 20 residue overlap, which resulted in 25,527 peptides from the allergenic proteins. The library also included the proteins of the B-cell antigens deposited in the IEDB by January 2018; this part of the library consists of 31,436 peptides. This library also contains roughly 40,000 peptides from phages that infect gut, pathogenic, and probiotic bacteria, as reported in Bourgonje *et al*. (47)

### Bacterial Libraries

In 2021, Vogl *et al*. (48) published a library consisting of peptides from bacteria primarily from the gut microbiota. The library contains potential antigens from the microbiome of healthy individuals from the metagenomics data of 953 stool samples in the personalized nutrition project, focused on secreted membrane, and motility proteins from bacteria this accounts for 60% of the library size. In addition to the metagenomic peptides, 10% of the peptides in the library come from bacteria that commonly cause gastrointestinal infections, 6% probiotic bacteria, 9% from bacteria previously reported to be coated by IgA antibodies, and 10% of the sequences come from the virulence factor database. In total, 28,668 proteins, 27,837 of which were microbiota proteins, were split into 64-mer peptides with 20-residue overlaps, resulting in 244,000 peptides. Angkeow *et al*. (49) developed the ToxScan. The library consists of nonviral proteins with the keyword word toxin (12,679 proteins) and the word virulence factor (2133 proteins) included in the UniProt database (as of November 2016). This resulted 14,430 unique protein sequences (436 archaeal, 7797 bacterial, and 5743 eukaryotic). These peptides were split into 56-mer peptides with 28-residue overlap for a total library of 95,601 peptides.

### Libraries of Model Organisms

Transgenic mice are a valuable tool for studying autoimmune diseases in a model system, and it is important that the model system resembles human disease. Two mouse-specific proteomic PhIP-seq libraries were established for mouse serum antibody profiling. Rasquinha *et al*. (50) in 2022, produced a mouse proteomic PhIP-seq library consisting of the mouse proteome (UP000000589) from the UniProt database (accessed on January 1, 2018) divided into 56-mer peptides with 28-residue overlap, resulting in 419,915 peptides. Rackaityte *et al*. (51) published in 2023 a murine PhIP-seq library with the (GRCm38.p5) reference proteome from NCBI (including all isoforms). The library consists of 62-mer peptides with a 19-residue overlap, resulting in a library of 482,672 peptides.

### Epitope Prioritization Library

All PhIP-seq libraries discussed thus far are based on whole protein sequences divided into tiles with certain residue overlaps, this results in inclusion if many peptides that does not contain antibody epitopes. If the sequence space of the proteins you want to include in your library by conventional methods is very large, the cost of making the oligos go up, and the pure number of individual phages that are included in the library becomes so large that you need to include such a high number of plaque-forming units to cover the sequence space that can affect the experiment. Liebhoff *et al*. (52) wanted to explore if the sequence number could be reduced by including only sequences with potential epitopes. Therefore, they developed and applied a machine learning algorithm called Dolphyn to reduce the number of sequences available to cover only the immunogenic peptides that are likely to include antibody epitopes by training the algorithm on sequences from so-called public epitopes (2). These public epitopes are present in multiple individuals and seem to contain sequence features that are important for germline-encoded antibody domains (11). They established a public epitope dataset (544 peptides) and constructed two initial libraries, one triple alanine scan and one library of peptides of different lengths (15–45 residues long), and their results showed that these public linear antibody epitopes are generally captured by 15-residue-long peptides. Based on this data, they trained the Dolphyn algorithm to find potential epitopes. The algorithm could distinguish epitopes from a test set with an area under curve (AUC) of 0.62. The algorithm was applied to a subset of phages found in the North American population from the gut phage database. They created a library consisting of three subsets of peptides, a standard 56-mer library with 28-residue overlaps, one Dolphyn subset consisting of 15-mer peptides representing the Dolphyn predicted epitopes, and a 56-mer subset containing three sequential Dolphyn predicted epitopes stitched with a GGGGS linker in between. The standard subset contained 23,745 peptides, the 15-aa Dolphyn subset contained 19,117 peptides, and the three sequential Dolphyn peptide subset contains 5266 peptides, for a total of 48,128 peptides. They tested this library on sera from healthy individuals and found that the Dolphyn-derived library increased the number of reactive peptides by from 10 to 31%. But the Dolphyn subset only recovered approximately 29% of the peptides from the conventional library; however, they also found some epitopes that were not recovered by the conventional sublibrary. The Dolphyn python package is available for download on Zendo (https://zenodo.org/records/10448728).

### Randomized Library

Finally, one could choose to use a more traditional randomized library to capture interactions through PhIP-seq, as Wu *et al*. (9) did so in a 2019 paper where they discovered potential biomarkers for systemic Lupus erythematosus (SLE) using a commercial randomized Ph.D-12 library from New England Biolabs. This library is also based on the M13 phage system. The selected peptides were then bioinformatically mapped to proteins with similar sequences. With that same library and similar selection system (AbMap), the group determined the specific linear epitope for more than 100 mAbs (53).

### Progression of Library Design

Over time, the peptide lengths of PhIP-seq generally increased from the initial 36-mer peptides (4) to the newer 54- to 64-mer libraries (39). This has happened as the quality of the oligo arrays of longer oligos has improved and are now at a point where they reach the length where next-generation sequencing lengths become an issue (54), both conventional NGS and oligo pool synthesis caps out at around 300 bp. Recently, Liebhoff *et al*. (52) used much shorter peptides in a stitched fashion after carefully determining the lengths of public epitopes. The advantages of libraries consisting of longer peptides are that they have the potential to capture antibody interactions based on conformational epitopes, and the advantage of libraries with shorter peptides is that linear epitopes can be resolved more easily. The length of the peptide influences the number of sequences in the library, where a library of longer oligos and fewer sequences is less expensive than a library of shorter oligos and more sequences in general, even though some suppliers price oligos of different lengths differently. Another risk with having longer oligos is the error rate increases with the length which means that a library with longer peptides will have a larger portion of phages displaying incorrect peptides. Many papers describing or using these libraries have utilized the PhIP-Seq method to construct smaller mutational and truncation libraries from peptides of interest to determine epitope bounds and residues important for recognition (2, 11, 34, 37, 52, 55).

### PhIP-Seq Library Construction

After careful bioinformatic curation of the proteins of interest be divided in to peptides and incorporated into the library, the peptide sequence is reverse translated into DNA using codons that are optimized for *E. coli* expression. In addition to the coding sequence, restriction sites for cloning were added to the 5′ and 3′ ends need to be added. The restriction sites used for cloning need to be removed from the coding sequence through silent mutations. If peptides of different lengths are used in the library, additional bases are added after the stop codons to make oligos of unified lengths (4, 56). These DNA sequences are then synthesized by a company and delivered as an oligonucleotide library pool. The pool is then PCR amplified and cloned into a T7 phage linear vector as a C-terminal fusion to the 10-3b protein of the phage. This will display 5 to 15 copies of the peptide per phage particle. After ligation, the library is packed into phage particles *in vitro* and then amplified in bacteria to form phage particles with displayed peptides. To increase the likelihood that the phage library coverage is sufficient to cover all sequences, there needs to be an oversampling of 100 to 1000 times as many plaque-forming units as sequences in the library (4, 34, 56). After the physical phage library is constructed, the library should be sequenced to determine its coverage and quality. Typically, the depth of sequencing ensures over 90% coverage of full-length correct inserts, and between 50 and 75% of all sequences are typically of the right length without substituting mutations, deletions, frameshifts, or premature stop codons. The frequency of sampled peptides for approximately 80% of the sequences is within one log_10_ of the mean peptide frequency for a successful library (4, 34). It is important that the library is fairly uniform to not introduce bias based no frequency, since the selection is typically one round.

### PhIP-Seq Selection

PhIP-seq selections can be used to analyze multiple samples in parallel; therefore selections are usually performed in a 96-well format. The antibody sources, for example, serum (56), plasma (57), cerebrospinal fluid (CSF) (58), or aqueous humor of the eye (59) are diluted and normalized between selections and mixed with the phage library and allowed to bind in solution. Itell *et al.* tested if dried blood stains can be used as the antibody source in PhIP-seq. They compared plasma and dried blood stains of patients and showed good correlation between the two systems (57).

After binding, the immunoprecipitate is pulled down using magnetic beads typically with protein A/G. However other methods have been used for IP, for example, mouse anti-human immunoglobulin E (IgE) and anti-human immunoglobulin G (IgG) have been used to test IgE- and IgG-specific epitopes in peanut allergies (3), and goat anti-human kappa and antihuman lambda antibodies have been used to test different epitopes discovered between different types of antibody light chains (11). After incubation, the magnetic beads (containing antibodies and bound phages) are pulled down using a magnetic stand, the liquid is removed, and the beads are washed repeatedly to remove unbound phages. Typically, PhIP-seq selection involves one round of selection, so after the wash step, the beads are suspended in ddH_2_O and heated to break the phage, after which the DNA is released and PCR amplified in a two-step PCR adding amplicons and then individual barcodes, pooled and cleaned up *via* gel extraction and purification and sent for NGS. After pooling before gel extraction an optional third round PCR can be performed for clearer band on the gel (56).

Using one round of selection requires large oversampling of the library to ensure that true binders can be selected over the background (2, 4). Multiple rounds of selection have been performed in some studies in which the phage is resuspended in LB and allowed to infect bacteria for *in vivo* amplification (34, 35). Unlike traditional phage display, there is no preselection in PhIP-seq. A selection should include controls of no library added in the well and mock-IPs where no serum is added. These mock-IPs are important for the statistical analysis described below.

## Evaluation of Results

After sequencing, the sequences are demultiplexed and aligned to the library *via* bowtie algorithm to identify what peptides and proteins are found. The sequences are also sorted by barcode for correct selection. Some sequences will be represented by hundreds or thousands of reads, and some sequences will have fewer reads. The selection is not specific enough to only return peptides with epitopes binding to the antibodies, some phage that has been bound to the plastic or to the magnetic beads will stick around and get sequenced (4, 60). Using raw read counts is usually not sufficient for identification of hits due to differences in peptide counts in the input library and because nonspecific binding in the experiment is not random, but some peptides exhibit nonspecific binding more than others. Instead of the raw number of reads per peptide, statistical analysis needs to be performed for hit identification. Currently, there are two major models for performing statistical analysis: the generalized Poisson distribution and the Z score algorithm (4, 60) and several papers have used individual statistical analysis like gamma poison (61) Recently, a third method, Bayesian enrichment estimation, was published (62). This review will briefly review the different statistical models, but for a more comprehensive review, see Filimonova *et al*. (30).

### Statistical Methods for Hit Determination

In the original PhIP-seq paper, Larman *et al*. (4) struggled with a high background of library phages in the NGS data from immune-precipitated phages (83% of the input library) to distinguish true hits from background, they employed a method adapted from RNA-seq analysis calculating *p* values for each sample peptide, compared to reads in the input, based on generalized Poisson distribution and then compared the log_10_ *p* values of two experimental replicates to find a cut of peptides enriched in both replicates. The method can be summarized as count value Yi ∼ Generalized Poisson (λ(Xi), θ(Xi)), where λ(x) = a x + b and θ(x) = c are empirically fitted through linear regression of λ, θ using peptide counts x (56). This method for hit identification has been used in a number of papers (2, 5, 33, 39, 46, 47, 48, 63, 64). In 2018, Yuan *et al*. (60) in a preprint proposed the use of a Z score enrichment algorithm, which is based on running a number of mock-IPs with the input library with only the pull-down magnetic beads present and no sample antibodies. The utilization of mock-IPs as the reference for determining the background makes more intuitive sense since it represents phages that are captured in the same way as in sample IPs, same type of wells in the plate, with the same blocking agent and same magnetic beads. To reach saturation of background phages, a number of replications of the mock-IP must be performed, which can be different depending on the library used. The sequencing results from the samples and mock-IPs were converted to reads per million (RPM) values. The sample RPM for a peptide is then compared to the RPM from the mock-IPs, and the peptides can then be assigned a Z score, which is how many SDs away from the mock-IP value the specific peptide has (60). The calculation of the Z score for one peptide can be described as follows: Z score= (RPM in sample-mean RPM mock-IPs)/SD in mock-IP (65). After calculating a Z score, a cut-off of a Z score that would be considered an enriched peptide needs to be set. This method has been employed in a number of recent papers (3, 37, 44, 51, 55, 60, 65, 66, 67, 68, 69, 70, 71, 72, 73, 74, 75, 76). Using this equation, outright runs the risk of having zero reads of the peptide in the mock-IP which would result in the SD to be 0 and a Z score not be calculated, this is solved by binning peptides of similar read counts in the mock-IP together as described in the supplemental methods of Mina *et al*. (66). Finally, in 2022, Chen *et al*. (62) published another method to detect enriched peptides in PhIP-seq data using Bayesian enrichment estimation. This method is based on edgeR that is a model for negative binomial distribution (77). This method of data analysis also relies on experimental mock-IPs to determine hits from the background at a rate similar to what is needed for Z score analysis. Regarding the advantage of this method, the authors argue that it is more sensitive, meaning that it can find more true hits of peptides with lower enrichment. In this method, the read counts of the sequences in a sample are modeled as a binominal distribution. This enables a fit of a Bayesian hierarchical model; from these fit, posterior probabilities of enrichment and estimated fold changes of the samples compared to mock-IPs can be derived. The system identifies superenriched peptides and removes them from the analysis to increase the sensitivity for peptides of lower enrichment. To test the false positive discovery rate, one can run the different mock-IPs against each other. The method uses a Markov chain Monte Carlo sampler, which computationally might be time consuming. A detailed description of the statistical method is published in Chen *et al*. (62) and an R-based software package “Bayesian Enrichment Estimation in R” were developed by Chen *et al.* (78), this package also runs edgeR for PhIP-seq analysis. To date, there few PhIP-seq papers published using Bayesian enrichment estimation as the hit determination method (79), but some more papers have used the edgeR negative binominal distribution (40, 45, 49, 50, 52, 80, 81, 82). For studies using two rounds of selection mainly from the DeRisi lab, a simpler statistical analysis based on fold change over mock IP have been utilized. Starting with normalizing the data usually to reads per hundred thousand (rpK) and then dividing the RpK in sample/mean RpK in mock IP (1, 34, 35, 51, 55, 58, 69, 72, 83, 84, 85, 86, 87). There are several computational packages that are able to do parts of these analyses most of them no longer maintained but one package that have all of the general analysis pipelines including edgeR and Bayesian Enrichment Estimation in R is phippery (88).

### Applications of PhIP-Seq for the Study of Circulating Antibodies

PhIP-seq is increasingly used in immunoproteomics to answer a multitude of different queries. Here, we present an overview of different use cases where PhIP-seq has been used to understand infection history, allergy, and autoimmune disease and how it can be utilized for biomarker discovery. The specific library used in the study can be found in Table 1.

### Public Pathogen Epitopes

By investigating viral epitopes of the human immune system, we can gain greater insight into how the immune system functions and evolves with infection history, which can aid in the diagnosis of infectious disease and can be used to associate prior infection with other diseases, for example, cancer. In 2015, Xu *et al*. published the first paper using VirScan library containing peptides from proteins of common infectious viruses. Nearly 600 individuals were tested, which at the time was the largest PhIP-seq cohort, on average samples had antibodies against ten viral species and antibodies against 84 species was found in at least two samples. Several samples was found to share the same epitope from viral proteins, these shared epitopes (public epitopes) (2) were further determined through saturation mutagenesis. The paper nearly doubled the number of known viral B-cell epitopes. Shrock *et al.* investigated the idea of viral public epitopes from Xu *et al*. further and showed that antibodies against the same public epitope in different humans used the same type of light chain (kapa or lambda) and through saturation mutagenesis of a subset of public epitopes and identified residues critical for recognition. They further sequenced B-cell receptors recognizing specific minimal epitopes in influenza A or Epstein‒Barr virus (EBV) and found striking similarities between the B-cell receptor sequences for the light chain V gene segment, suggesting that this gene is encoded in the germline. They also established a motif (GRAB) within the antibody responsible for binding lysine residues often present at the edge of public epitopes (11). Venkataraman *et al.* studied heritability of EBV epitopes using VirScan and single nucleotide polymorphism (SNP) genotyping on a twin cohort. They reported heritability of the viral epitope antibody response for 107 EBV epitopes and could show that some epitopes were associated with specific major histocompatibility complex (MHC) class II locus (74). In a study using libraries containing bacterial and viral allergenic peptides, Andreu-Sánchez *et al*. found associations between reactivity to specific peptides had strong association with specific SNPs in the MHC class II locus (64). Olin *et al*. also found associations with antiviral response and specific SNPs in MHC class II and also found an association between higher response to rhinoviruses in individuals who smokes (89). Pou *et al.* used VirScan to determine the maternal antiviral antibody repertoire in new-born preterm babies and babies carried to term (90).

### Immunosuppression and Targeted Anti B-Cell Therapies Effect on the Antibody Repertoire

B-cell malignancies and autoimmune diseases require targeted therapies against B cells in recent years chimeric antigen receptor T-cell (CAR-T) therapies have been established to kill B-cell malignancies. Hill *et al*. investigated how anti CD19-CAR-T cells designed to kill CD19+ B cells affect the antibody reactome against viruses. They showed that anti-CD19 CAR-T therapy for B-cell malignancies had little effect on virus specific epitopes and a modest decrease in total IgG (91). Bodansky *et al*. investigated several B-cell–depleting therapies and confirmed that CD19 CAR-T along with CD20-depleting rituximab had little effect on the antibody repertoire against human epitopes but anti BCMA CAR-T therapy (marker specific on plasma cells) had a profound effect on epitope recognition. Bender Ignacio *et al*. used VirScan to test patients undergoing hematopoietic cell transplantation and reported that the antibody response was more similar to that of the donor than to that of the self at 100 days after transplantation, but was more similar to that of the self at 365 days after transplantation (92). Isnard *et al*. reported that immunosuppressive treatment after kidney transplantation had limited effect on antibody reactivity against viral infection (93).

### Viral Epitopes Association with Inflammatory Diseases

Bjornevik *et al*. used serum samples collected through the department of defense serum repository in the United States from people who developed multiple sclerosis (MS) (801) and healthy controls (1,566) and tested them for EBV infection over a 20-year time period. All but one of the MS patients were positive for EBV or became EBV positive during the study before the onset of MS symptoms. Thirty MS patients and matched controls were subjected to PhIP-seq, which revealed that the strong signal of EBV peptides differed between patients and controls both in preonset and postonset samples (94). In a follow-up study, Cortese *et al.* reanalyzed the results from Bjornevik *et al.* and found although there are differences in the level of antibody response between MS and controls there are no peptides that were specifically enriched in the MS population but higher anti-EBNA-1 response was associated with MS. Enose-Akahata *et al*. confirmed that increases in serum and CSF EBV antibodies are associated with MS (95). A recent study revealed an association between increased titers of anti-EBV and anti-CMV at 6 months after onset of long coronavirus disease 19 (COVID-19) patients compared to controls (96). Hasan *et al.* demonstrated that exposure to herpes simplex virus-1 is associated with obesity in an adult population but not in children (63). Wang *et al*. showed that patients with more severe COVID-19 had higher levels of antibodies to a wide range of viruses, suggesting that severe COVID-19 stimulates memory B cells to produce antibodies against unrelated infections (97).

### Using VirScan to Investigate Ongoing Infections

Galardi *et al.* used VirScan to associate pediatric encephalitis in Myanmar with enterovirus herpes simplex virus-1 and respiratory syncytial virus infection (1). Leon *et al*. increased the rate of enterovirus A71 diagnosis in a pediatric brainstem encephalitis outbreak in Catalonia using VirScan (98). Hawes *et al*. used VirScan and a human peptidome library to study antibody reactivity in the serum and CSF of HIV-positive patients’ neuro-symptomatic and asymptomatic antiretroviral therapy escape (86). Ruhs *et al*. used VirScan to study the serological profiles of bats to demonstrate its use for viral surveillance of emerging zoonoses (76). VirScan can also be used in case studies of specific patients with complex diseases. Johnson *et al.* studied a patient with progressive dementia and found enrichment of dengue viral antibodies in CSF compared to serum (99). Stoddard *et al*. produced a library with seven hCoVs including SARS-CoV-2 showed that people with no SARS-CoV-2 exposure had little cross reactivity with SARS-CoV-2 peptides but people who have had COVID-19 had broad cross reactivity with other hCoVs (38).

### Bacterial Epitopes and Their Role in Disease

Vogl *et al*. created a library of bacteria, both probiotic, pathogenic, and bacteria present in the gut microbiota (48). They discovered a large set of both public and private epitopes to bacteria that are associated with age and sex and that are stable over time (48). The ToxScan library consists of peptides from proteins with mainly bacterial origin and some with eukaryotic parasite origin. This library was used to investigate epitopes in patients with Chron’s disease, juvenile dermatomyositis, and healthy cohort. They also found that bacterial epitopes are stable over time. They found that anti flagellin antibodies was associated with juvenile dermatomyositis and Chron’s disease (49).

### Discovering Epitopes with Implications for Vaccine Design

As described above, two parasite-specific libraries have been produced and used for PhIP-seq experiments, namely*, Plasmodium falciparum* (44) and *Schistosomiasis mansoni*^8^. In the Plasmodium study, plasma from adults and children from high- and moderate-transmission regions in Uganda and from American blood donors were used as controls. A total of 9927 seroreactive peptides from 1648 parasite proteins were identified, 952 of which have not been reported in other high-throughput studies. Importantly, antigens in regions with short amino acid–residue repeat regions only elicit a strong antibody response in areas of high transmission and are short-lived responses, which have implications for vaccine design (44).

The *schistosomiasis mansoni* library was tested in rhesus macaques through infection and clearance. Antibodies against several stages of the life cycle were found throughout the progression of the disease. Peptides from two *schistosomiasis* proteins, SmCatB and SmAE, were used to immunize mice and elicited an antibody response and reduced the total parasite burden postimmunization infection; these results have implications for vaccine design (8). Yalcin *et al.* used the SARS-CoV-2-specific library described by Shrock *et al.* (37) to follow subjects trough first, second, and booster vaccination with the BNT162b2 (Pfizer/BioNTech) vaccine (61) and found that the breadth of epitopes recognized was greatest after the second shot, but boosting resulted in a greater response. Sabatino Jr *et al*. (100) investigated the immune response to COVID-19 vaccination (Pfizer, Moderna, and Johnson & Johnson), with the 7 CoV library described by Zamecnik *et al*. (36) in patients undergoing different immune-modulating treatments for MS, which showed a narrowing of the breath of epitopes in patients undergoing treatment compared to nontreatment patients and healthy controls. The same library was used by Prahl *et al*. to investigate anti spike protein antibody transfer to the child after SARS-CoV-2 vaccination of during pregnancy (101). Mina *et al.* used VirScan to measure antibody reactivity before and 7 weeks after measles infection of children. They show an 11 to 73% decrease in the antibody repertoire after infection, which is not observed in children receiving MMR vaccination, underscoring the importance of childhood measles vaccination (66).

### Autoantibodies, Immune System, and Diseases

Host-specific PhIP-seq can be used to study immune system dysregulation. The original Larman *et al*. study from 2011 included three case studies of patients with paraneoplastic syndrome in which epitopes were found from proteins expressed in the brain (GAD65, TRIM9, TRIM67, and the hypothetical protein LOC26080) that are likely to contribute to the disease. O’Donovan *et al*. also investigated paraneoplastic patients who had anti-Yo and anti-Hu syndrome and confirmed and discovered antigens for those syndromes as well as described the epitope on a residue-level resolution for anti-Hu nELAVL-specific antibodies (34). In a case study using PhIP-seq of a patient, anti-Yo antibodies were detected regardless of a previous false negative diagnosis (102). Another case report of two patients with potential paraneoplastic syndrome revealed autoantibodies against βIV-spectrin using PhIP-seq (103). Ishikawa *et al*. found KLHL11 autoantibodies in a case report of a man with paraneoplastic syndrome associated with metastatic seminoma (71). In a case report involving a patient with well-controlled HIV infection presenting with meningoencephalitis, Bartley *et al*. reported autoantibodies against ankyrin G (73). In 2013, Larman *et al*. performed the first large screen using human peptidome PhIP-seq in 298 independent samples and identified autoantibodies for MS, type 1 diabetes, and rheumatoid arthritis (5). Song *et al.* investigated the CSF of COVID-19 patients with neurological symptoms and found reactive epitopes in three proteins present in both PhIP-seq and IP coupled to mass spectrometry experiments (UHRF1BP1, NUAK1, and DBN1) (58). Mandel-Brehm *et al*. used PhIP-seq to detect autoantibodies to Perilipin-1 in a subset of patients with acquired generalized lipodystrophy (104). Mutations in the AIRE gene give rise to autoimmune polyendocrine syndrome type 1 (APS1), which is an autoimmune disease that can affect multiple organs in the body. Vazquez *et al*. used PhIP-seq to identify novel autoantibodies that target specific organs affected in patients, such as anti-KHDC3L autoantibodies, which target ovaries and are associated with primary ovarian insufficiency, and anti-RFX6 autoantibodies, which target the gut and are associated with diarrheal-type intestinal dysfunction in patients with APS1 (67). Davoudi *et al*. screened 11 patients with autoimmune retinopathy matched with healthy controls and found case-specific autoantibodies in several of the patients (105). By using peptidyl arginine deiminase to citrullinate arginines in a human peptidome library, Román-Meléndez *et al*. found that PhIP-seq can identify antibody antigen pairs that depend on the posttranslational modification citrulline, which is important for antigen discovery in rheumatoid arthritis (106).

Rackaityte *et al*. and Rasquinha *et al*. both created mouse peptidome PhIP-seq libraries (50, 51). Rackaityte *et al*. used their library to test seven genetically distinct mouse lines that serve as model systems for immune function and autoimmune diseases and found autoreactivity similar to that observed in their human disease models, validating their usefulness (51). Rasquinha *et al*. used their library with plasma from mice that had virus-induced myocarditis, which is a model system used to study the disease progression of myocarditis into dilated cardiomyopathy, and found autoantibodies to 32 peptides from 25 proteins not previously reported, most notably COA4, which was also found when screening the human peptidome against the same mouse sera (50).

### Identifying Potential Epitope Biomarkers for Disease

Identifying biomarkers that can be used in serological tests to diagnose disease or differentiate treatments or outcomes is valuable for clinical use. PhIP-seq is a tool that is well suited for identifying biomarkers that are relevant for autoimmune and neurodegenerative diseases, as well as cancer and infection. Our group in Wu *et al*. used a randomized phage library to identify four peptides that had high diagnostic power for differentiating patients with SLE from healthy controls (9). Liu *et al*. utilized VirScan to detect a viral exposure signal that was associated with hepatocellular carcinoma and used it prospectively in at-risk patients to identify patients who were going to develop cancer, and their viral exposure signal biomarker was superior to that of the alpha-fetoprotein test (107). The same group subsequently studied a larger cohort of patients with liver diseases and healthy controls and reported that nonhepatitis virus, serological viral profiles associated with liver disease, and liver disease protection can serve as biomarkers for at-risk populations (70). Mitchell *et al*. used VirScan to study antibody reactivity in pediatric patients with severe acute hepatitis of unknown etiology and reported a strong response to adeno-associated virus 2 (108). Eshleman *et al*. studied the evolution of the antibody response in patients infected with HIV *via* VirScan and based on the differences in virulence between early- and late-stage HIV infection, and they designed a peptide that can be used as a biomarker in cross-sectional studies that can distinguish early-stage infection from late-stage infection (55). Yaffe *et al*. made a HIV envelope–specific PhIP-seq library and found that infants who acquired HIV had better survival if they had passively acquired antibodies against constant region 5 of the envelope protein through breastfeeding (43). Zamecnik *et al*. used a SARS-CoV-2–specific PhIP-seq library and coupled the results with a phage microarray to identify epitopes that could be used as biomarkers for COVID-19 infection and argued that this pipeline is well suited for rapid biomarker identification in emerging pandemics (36). Shrock *et al*. used different SARS-CoV-2–specific PhIP-seq libraries to identify the antibody response to COVID-19 infection and found that the strength of the antibody response correlated with the need for hospitalization; moreover, they found that peptides from PhIP-seq could discriminate COVID-19 infection and created a Luminex-based serological assay that performed as well as gold standard ELISAs for COVID-19 (37). In a follow-up to the APS1 study discussed in the previous paragraph, Vazquez *et al*. established methods for a scaled PhIP-seq protocol that can test 800 samples in parallel and used them to generate large datasets of antigens for APS1, IPEX, RAG1/2 deficiency, Kawasaki disease, multisystem inflammatory syndrome in children, and mild and severe forms of COVID-19 and employed machine learning to construct models that can predict disease status and individual antigens that could serve as biomarkers for these diseases. As well as finding autoantigens shared between diseases could shed light on the causes of those diseases (69). Upadhyay *et al*. used human peptidome PhIP-seq against a cohort of patients with interstitial lung disease (ILD) and healthy controls. Autoimmune ILD can affect people with various connective tissue disorders, and it is important to establish autoimmunity in ILD for prognosis and treatment. These authors validated cadherin-related family member 5 as a potential biomarker for autoimmune ILD in patients with rheumatologic disorders (75). Mandel-Brehm *et al*. found ZSCAN1 autoantibodies in the CSF of seven of nine patients with tumor-associated rapid-onset obesity with hypothalamic dysfunction, hypoventilation, and autonomic dysregulation and in 0/125 controls. This validated autonomic dysregulation as a paraneoplastic syndrome, with the ZSCAN1 autoantibody as a putative biomarker (65). Dubey *et al*. found autoantibodies to caveolae-associated protein 4 (cavin-4) in eight out of 10 patients with immune-mediated rippling muscle disease, which can be used as a serological biomarker in this disease (109). Fleischer *et al*. found autoantibodies that could differentiate SLE patients that have had myocardial infarction with systolic dysfunction from SLE patients that had not had a heart attack (110). Using the human peptidome library Hosono *et al.* found that having autoantibodies to transcription factor Sp4 was protective against dermatomyositis patients with anti-TIF1γ autoantibodies (111). Bodansky *et al*. found autoantibodies toward an epitope in SNX8 in patients that gotten multisystem inflammatory syndrome in children from SARS-CoV-2 infection that overlapped with an epitope in the SARS-CoV-2 N protein (83). Zamecnik *et al*. found an epitope signature present in several human proteins that overlaps with pathogens (including EBV) that some patients who develop MS have autoantibodies against even before onset of symptoms (84).

### IgE and IgG Antibody-Epitopes in Patients with Allergy

Allergy is a dysfunction of the immune system, where the body produces IgE antibodies against harmless particles from food and other sources. IgE binds to mast cells and basophils *via* the FcεRI receptor, crosslinking of the FcεRI by the allergen binding to a pair of IgE antibodies leads to the release of histamine that can trigger anaphylaxis (112). Chen *et al*. used a peanut-specific PhIP-seq library to investigate the immune response to peanut allergy in people receiving oral immune therapy (OIT) for peanuts and healthy controls (3). Both the IgG and IgE responses to those allergens were assessed. Nonallergic individuals had no IgE antibodies reactive to any of the peanut epitopes and low IgG reactivity, and allergic patients had some public and private IgE reactive epitopes and higher IgG reactivity than healthy controls. The epitope recognition ability of IgE and IgG was similar for allergic patients, but that of IgG was lower. After OIT, the IgG antibodies recognized mote peanut epitopes with greater overlap with IgE and additional IgG-specific epitopes. The number of IgE-specific epitopes remained the same. The abundance of IgE in the serum was deemed to be lower based on the epitope-specific Z score before and after OIT. They also postulated that the increase in IgG can block IgE binding and subsequent FcεRI receptor activation (3). Monaco *et al*. used a library representing all known protein allergens to study patients with a clinical diagnosis of wheat allergy matched with healthy controls and OIT (45). They also measured specific IgE and IgG responses in their subjects. For people with known wheat allergies who are “sensitive” and healthy controls, the study revealed minimal reactivity to IgE for healthy and sensitive individuals and expansive reactivity for allergic individuals. For IgG, they found that high reactivity to similar epitopes for allergic people was lower for sensitized individuals and minimal for healthy controls. Studying OIT for wheat, they also found, as in the peanut study, a decrease in the Z score for IgE peptides and an increase in the score for IgG peptides compared to those of the placebo group (45). Leviatan *et al*. used a different PhIP-seq library representing known allergens to study the IgG and IgA response in individuals without allergies (46). This study tested a large cohort of 1003 individuals representing the Israeli population (which included some people with allergies). They found IgG response to specific epitopes from common food allergenic proteins was found in up to 50% of individuals, especially epitopes in dairy proteins. They also found that IgG and IgA bound distinct epitopes (46).

### Machine Leaning and Bioinformatics Analysis of PhIP-Seq

PhIP-seq results in large cohorts offer an opportunity to further investigate them with bioinformatics approaches and machine learning. The anti-viral antibody response deconvolution algorithm is an algorithm based on hits from VirScan that provides estimates of previous viral infections based on sequence alignment between the hits and all human viruses (68). This method was able to improve the rate of diagnosis of herpes virus–induced viral encephalitis by 44% and could associate prior enterovirus infection with the development of disease in a cohort genetically predisposed to type 1 diabetes. Upadhyay *et al*. trained a random forest classifier to determine what hits would best discriminate between samples and controls of autoimmune ILD (75). Bourgonje *et al*. used the human microbiota library (48) together with the library allergens and B-cell antigens included in the IEDB and common gut phages (46) to study bacterial epitopes in patients with inflammatory bowel disease (47) compared with healthy controls. They found differences in 373 epitopes between healthy controls and patients with inflammatory bowel disease (202 overrepresented and 171 underrepresented). Of those 373 epitopes that showed differences, 205 were exclusively associated with Crohn’s disease (CD), 104 with ulcerative colitis (UC), and 64 with shared epitopes. Using machine learning on the antibody epitope repertoire, the model was shown to discriminate between UC or CD patients and healthy controls (AUC 0.8–0.89) and to moderately differentiate between CD patients and UC patients (AUC 0.68) and through recursive feature elimination found ten epitope signatures that was as good as the whole dataset for classification (47). Klompus *et al*. created a human and animal coronavirus virion library and tested people who were exposed to SARS-CoV-2 and unexposed controls. These researchers showed substantial antibody cross-reactivity between hCoV and aCoV peptides and showed that mAbs from recovered COVID-19 patients can have broad cross-reactivity with hCoV and aCoV They also developed a machine learning algorithm that could distinguish people who have had COVID-19 from controls based on the cross reactivity with an AUC = 0.984 using the whole library and 0.957 using the animal CoV peptides (39). Several studies have employed machine learning to match PhIP-seq results with COVID-19 infection (37, 87, 97). In a screen of a cohort of patients with autoimmune hepatitis, Klepper *et al*. found several novel autoantibodies for instance DIP2A were found to be antigens that share a nine aa epitope with a peptide from human herpes virus 6B, which could be a potential origin for some cases of autoimmune hepatitis, and used logistic regression to predict autoimmune hepatitis *versus* healthy controls with an AUC of 0.65 (72) Bennett *et al*. in two papers, described the antibody response to Kaposi sarcoma-associated herpesvirus in people who developed Kaposi sarcoma compared with those who did not and the effect of HIV infection has on the antibody response (113, 114). In the second paper, they used machine learning to classify people who develop Kaposi sarcoma from people who are asymptomatic carriers with an AUC of 0.967 (114). In a follow-up study about APS1 mentioned above, Vazquez *et al*. used logistic regression to classify patients with APS1 from healthy control with an AUC of 0.95 (69). Do *et al*. trained XGboost models to distinguish between patients with hepatocellular carcinoma or intrahepatic cholangiocarcinoma *versus* healthy controls and hepatocellular carcinoma *versus* chronic liver disease, with AUC of 0.778 and 0.693 and 0.57, respectively (70).

### Limitations of PhIP-Seq

Using phage display libraries to perform IP coupled with sequencing carries limitations and further method development could reduce these limitations. First, in the PhIP-seq sequencing results, there is a large quantity of background peptides (4). The background peptides are due to unspecific binding to materials in the experiment and persist through the wash steps and being read in the sequencing results making it hard to distinguish between true binders and background peptides, analogous to the CRAPome for affinity purification-mass spectroscopy experiments (115). Each library has unique phages that tend to be included in the background more often than others. This means that you bioinformatically and statistically have to remove or reduce this noise. This leads to the use of additional controls for mock-IPs (60, 62). The background is also a problem in the NGS sequencing step because it requires deeper sequencing to sort hits from the background (53). If the background is reduced, it would mean that more true binders can be included for sequencing, which would enable larger studies and reduce costs. It is also possible that true binders are removed in the cut-offs, especially if the binding antibody is less frequent in the sample. Since PhIP-seq generally involves one or two rounds of selection without preselection, the elimination of “sticky” phages can be difficult, but we think that there is room for method development in the experimental part of PhIP-seq that could address this issue as laid out in future perspectives below. Second, *in vivo* proteins undergo posttranslational modifications (PTMs), such as side chain modifications, for example, phosphorylation, citrullination, methylation, and acetylation, as well as larger PTMs, such as glycosylation (both by single sugars and poly-glycosylation with branched chains) (116) and covalent attachment of small proteins (such as ubiquitinylation or SUMOylation) (117). None of these PTMs are inherently incorporated in PhIP-seq since the peptides are expressed in bacteria and therefore will be absent during selection. In phage display, this can be addressed to some extent by using enzymes to carry out PTMs of the peptides on the phage (106, 118). Alternatively, unnatural amino acids can be incorporated into phage display libraries through the use of suppressor tRNAs (119). Alternatively, PTMs could be mimicked by substitution to one of the other of the 20 natural amino acids (118, 120). Using enzymes does not guarantee full coverage of the PTM or that the PTM is incorporated at the same places as *in vivo*, and suppressor tRNA can lead to premature termination and slower peptide expression, leading to underrepresentation of phages presenting the PTM. Using PTM mimetic amino acids might not be well suited for antibody epitope recognition. Another limitation is the discovery of antibody–antigen interactions that are dependent on structural epitopes. The longest PhIP-seq library is 90-aa long, which is longer than the shorter modular protein domains that can fold and function by themselves (121). Having longer peptides increases the opportunity for peptides to form their native secondary structure, which can be important for antibody recognition (122). The notion that the longer peptides in PhIP-seq have a structure was bolstered by Vogl *et al.* who found peptides from staphylococcal protein A in their selections with the microbiota library (48), which is an interaction between a structural domain of the staphylococcal protein A and the fragment crystallizable region of antibodies. More strikingly, the original PhIP-seq study by Larman *et al*. with a library consisting of 36-mer peptides revealed an epitope that was not reactive in immunoblotting of denatured proteins but was able to be immunoprecipitated under nondenaturing conditions, suggesting a dependence on secondary structure for recognition (4). Not all antibody binding is dependent on secondary structures spanning long sequences; short epitopes do not bind linearly but bind in conformations with defined secondary structures (123). However, even 64-mer peptides are unlikely to capture a large part of discontinuous conformational epitopes (48, 61). When searching an in-house list of 1158 structural epitope–Ab interactions from the protein data bank for the distance between the first and last residues of the epitope that participate in the interaction, 33% of the structures contained epitopes with a primary sequence length shorter than 90 residues, and 10% had a length shorter than 50 residues. Not all of those structural epitopes would likely be captured by PhIP-seq because they depend on correct folding on the surface of the phage for structural epitopes to be captured, and we speculate that many peptides on the phage would not be capable of correct folding because they start and/or stop in the middle of what would be a structured region. It is evident from the results from several studies that PhIP-seq to date seems to generate many epitopes that have short motifs and can be defined to 4 to 14 aa (2, 3, 11, 34, 37, 51, 52, 64, 124). In the case of biomarker generation, shorter peptides can be good from a materials and cost perspective (9), but this is not optimal for other PhIP-seq applications. Last, if PhIP-seq is to become a routine avenue for experimental immunological laboratories without specific expertise, there needs to be a more standardized accessible pipeline for data analysis that could be integrated into bioinformatics applications with graphical interfaces.

### Future Perspectives

In this decade, PhIP-seq has become a very powerful technology for analyzing the antibody reactome and will and should be applied more widely in the near future, as we can see how it is becoming more widely used, with over 25 papers published on the subject in 2023 up from two studies in 2018. To reach its full potential for widespread use, we will see technology development and standardization of PhIP-seq, making PhIP-seq more approachable for the average lab. We anticipate experimental strategies for reducing the signal-to-noise ratio of the selections. One interesting approach that could be investigated for PhIP-seq is instead of the use of magnetic beads using nonfouling porous PEG hydrogel as the matrix for selection, as was recently demonstrated for aptamer selection (125). Mohan *et al*. (56) published a protocol for library design and selection, and we anticipate that there will be more efforts to make library design more standardized and streamlined. We also anticipate that the standardization of data analysis will make different PhIP-seq studies easier to compare and more approachable. For larger studies, there will be more automation of the experimental procedures, both using robotics and dedicated benchtop equipment, as in Vazquez *et al*. (69) A repository of standardized processed results as suggested by Tiu *et al*. (29) would aid in this comparison and would enable labs to perform meta-analyses on PhIP-seq data or use results that were not the top line focus of papers both to validate their own results and for further studies. A repository of PhIP-seq results can also be integrated into multiomics analyses, which has been done by Venkataraman *et al.* (74), Vogl *et al.* (48) and Andreu-Sánchez *et al*. (64) which is integrated into the larger LifeLines cohort (126). We anticipate that PhIP-seq will be more integrated as part of large cohort multiomics studies in the future because both biomedical research and funding are heading and because of the power of the PhIP-seq technique itself. As we have seen in recent years, more libraries are being published from several different laboratories. We anticipate that many novel libraries tailored to specific research questions, such as the anellome scan (40) or the peanut allerscan (3), will be created. The effort by Liebhoff *et al*. to reduce the sequence space of PhIP-seq libraries is an interesting approach and we anticipate that other approaches for reducing the sequence space will be made as prediction-tools for epitopes improve or designing tools that exclude sequences that are not likely to contain epitopes. One approach used for protein–protein interaction identification is to use only sequences from surface exposed disordered parts of proteomes (127, 128), similar approach might be of use also for PhIP-seq. We predict that PhIP-seq, both in multiomics studies and in more detailed disease focused studies, will be used to answer more fundamental questions related to basic biology and the immune system. The usefulness of PhIP-seq for biomarker identification for various autoimmune, allergy, and inflammatory conditions will drive more such discoveries. Clinicians will continue to use PhIP-seq data as part of case studies of difficult-to-diagnose individuals.

### Search Strategy and Selection Criteria

Data in the PhIP-seq part of the review were identified through searches of PubMed and Google Scholar using search terms of phage immunoprecipitation and sequencing, PhIP-seq, and VirScan. The review includes to our knowledge all published PhIP-seq libraries. Additional PhIP-seq specific papers were found in the references list for those publications. Preprints that are included in the review were all indexed by PubMed. References that are not specific to PhIP-seq was found through searches in PubMed using reference specific search terms or in reference lists of other included references.

## Conflict of interest

The authors declare no competing interests.
